# Monitoring and evaluating the impact of national school-based deworming in Kenya: study design and baseline results

**DOI:** 10.1186/1756-3305-6-198

**Published:** 2013-07-05

**Authors:** Charles S Mwandawiro, Birgit Nikolay, Jimmy H Kihara, Owen Ozier, Dunstan A Mukoko, Mariam T Mwanje, Anna Hakobyan, Rachel L Pullan, Simon J Brooker, Sammy M Njenga

**Affiliations:** 1 Eastern and Southern Africa Centre of International Parasite Control (ESACIPAC), Kenya Medical Research Institute (KEMRI), P.O Box 54840–00200, Nairobi, Kenya; 2 Faculty of Infectious and Tropical Diseases, London School of Hygiene and Tropical Medicine, Keppel Street, London WC1E 7HT, United Kingdom; 3 Division of Vector-borne and Neglected Tropical Diseases, Ministry of Public Health and Sanitation, P.O. Box 19982–00202, Nairobi, Kenya; 4 Development Research Group, The World Bank, 1818 H Street NW, Washington, D.C 20433, United States of America; 5 Children’s Investment Fund Foundation, London, United Kingdom; 6 KEMRI-Wellcome Trust Research Programme, P.O. Box 43640–00100, Nairobi, Kenya

**Keywords:** Soil-transmitted helminths, Schistosomiasis, School-based deworming, Monitoring and evaluation, Kenya

## Abstract

**Background:**

An increasing number of countries in Africa and elsewhere are developing national plans for the control of neglected tropical diseases. A key component of such plans is school-based deworming (SBD) for the control of soil-transmitted helminths (STHs) and schistosomiasis. Monitoring and evaluation (M&E) of national programmes is essential to ensure they are achieving their stated aims and to evaluate when to reduce the frequency of treatment or when to halt it altogether. The article describes the M&E design of the Kenya national SBD programme and presents results from the baseline survey conducted in early 2012.

**Methods:**

The M&E design involves a stratified series of pre- and post-intervention, repeat cross-sectional surveys in a representative sample of 200 schools (over 20,000 children) across Kenya. Schools were sampled based on previous knowledge of STH endemicity and were proportional to population size. Stool (and where relevant urine) samples were obtained for microscopic examination and in a subset of schools; finger-prick blood samples were collected to estimate haemoglobin concentration. Descriptive and spatial analyses were conducted. The evaluation measured both prevalence and intensity of infection.

**Results:**

Overall, 32.4% of children were infected with at least one STH species, with *Ascaris lumbricoides* as the most common species detected. The overall prevalence of *Schistosoma mansoni* was 2.1%, while in the Coast Province the prevalence of *S. haematobium* was 14.8%. There was marked geographical variation in the prevalence of species infection at school, district and province levels. The prevalence of hookworm infection was highest in Western Province (25.1%), while *A. lumbricoides* and *T. trichiura* prevalence was highest in the Rift Valley (27.1% and 11.9%). The lowest prevalence was observed in the Rift Valley for hookworm (3.5%), in the Coast for *A. lumbricoides* (1.0%), and in Nyanza for *T. trichiura* (3.6%). The prevalence of *S. mansoni* was most common in Western Province (4.1%).

**Conclusions:**

The current findings are consistent with the known spatial ecology of STH and schistosome infections and provide an important empirical basis on which to evaluate the impact of regular mass treatment through the school system in Kenya.

## Background

In Africa, an increasing number of countries have launched national Neglected Tropical Diseases (NTDs) Strategic Plans, with Kenya being among the first to launch its multi-year plan in November 2011. The main strategy of these plans in tackling NTDs in Africa is the delivery of preventative chemotherapy, including school-based deworming (SBD) to control soil-transmitted helminths (STH) and schistosomiasis. There are also attempts to integrate mass drug administration (MDA) for STH and schistosomiasis with other NTDs, including lymphatic filariasis and onchocerciasis.

The STHs, *Ascaris lumbricoides*, *Trichuris trichiura* and hookworms, are estimated to infect over 1 billion individuals worldwide and in 2010 caused 5.18 million disability adjusted life years (DALYs), while schistosomiasis contributes 3.31 million DALYs [1]. Chronic infections can have insidious effects on childhood development, including growth and cognitive development, whilst heavy infections may result in serious clinical disease. Both chronic and intense infections are most common in school-age children who are the natural targets for school-based chemotherapy programmes. Reaching the school-aged population is most effectively achieved via the school infrastructure, and SBD programmes have been shown to be a simple and cost-effective strategy to reduce the disease burden of STH [2]. In 2001, the World Health Assembly endorsed the WHA 54.19 resolution that urged countries to control morbidity due to STH infection through regular deworming of school-aged children, setting a target to deworm 75% of the school children [2]. In support of this resolution, an important recent development was the large scale donation of deworming drugs by pharmaceutical companies in 2010, with GlaxoSmithKline donating 400 million albendazole tablets per year and Johnson & Johnson donating 200 million mebendazole tablets per year. Concomitantly, there has been increased demand for government led SBD programmes and donors are willing to fund national programmes either as part of school health programmes or integrated NTD control programmes. As programmes are scaled-up there is a scientific imperative to monitor the efficacy of treatment and to rigorously document the impact of treatment on infection and health outcomes.

In Kenya, a national SBD programme was launched in 2009, with financial support from the Ministry of Education and technical support and drugs provided by Deworm the World and the Partnership for Child Development. The programme successfully treated over 3.6 million school-age children. This programme built on previous pilot programmes in Kenya [3-5], including a pilot school health programme in Mwea District in central Kenya supported by the Eastern and Southern Africa Centre of International Parasite Control (ESACIPAC) at the Kenya Medical Research Institute (KEMRI) and the Japan International Cooperation Agency [6]. In addition to the operational experience in implementing SBD programmes in Kenya, there exists a clear policy context, with school health policy and guidelines developed jointly by the Ministry of Public Health and Sanitation and the Ministry of Education [7,8]. In 2011, the national government programme (with technical support from Deworm the World) received five years of funding from the Children Investment Fund Foundation and in 2012, an estimated 4.6 million school children received albendazole treatment for STH infection (Government of Kenya, pers. comm.).

This paper describes the overall study design of the monitoring and evaluation (M&E) of the Kenya national school-based deworming programme and presents results from the baseline survey. Patterns of infection by age, sex and geography are also reported.

## Methods

### Study design

**Figure 1:**
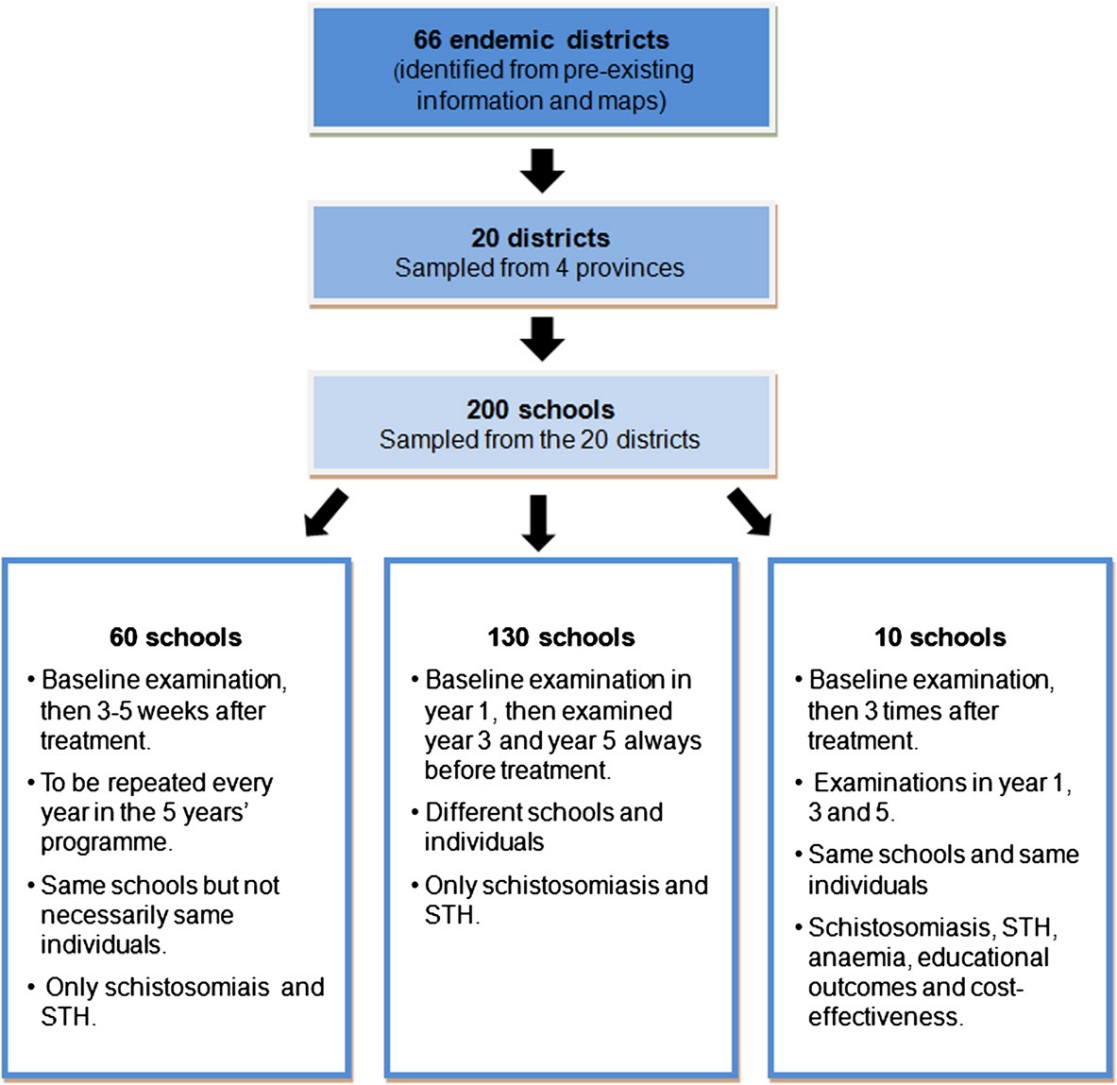
**Schematic of M&E programme design.** Three tiers of monitoring are conducted: i) a national baseline survey; ii) pre-post surveys, and iii) high frequency surveys.

The M&E includes a series of pre- and post-intervention, repeat cross-sectional surveys in a representative, stratified, two-stage sample of schools across Kenya. District stratification was based on both geography and anticipated infection prevalence. The programme contains three tiers of monitoring: i) a national baseline survey including 200 schools in 20 districts, which aims to establish an accurate national measurement of infection levels; ii) surveys conducted pre and post intervention (pre-post surveys), which monitor 60 of the 200 schools before and immediately after the deworming activity to evaluate reductions in infections that can be directly attributed to programme implementation; and iii) high frequency surveys in 10 schools, distinct from the 60 pre-post schools, at four time points in a single year, before, during, and after treatment (Figure 1).

Two hundred schools were examined at baseline and will be re-examined in year 3 and 5 in order to monitor long-term changes in worm infection at a national level both in terms of prevalence and intensity of infection. This sample size was chosen in order to be able to detect a five-percentage-point change in prevalence across years, assuming power *β* = 0.80 and test size *α* = 0.05, and considering the anticipated variance in prevalence. Sixty schools (a subset of the 200) will be surveyed every year for 5 years, before each treatment round to evaluate programme impact and 3–5 weeks post-treatment to evaluate treatment efficacy [2]. The same schools will be surveyed each year in the 60 pre-post survey schools, whereas in the remaining 130 schools, a different random sample of schools will be undertaken each year. In the 10 high frequency schools, a cohort of children will be followed-up longitudinally and assessed for haemoglobin concentration in addition to parasitological outcomes.

The 200 schools were selected based on the geographical distribution of population and STH endemicity. Based on available data and predictive maps [9,10], STH was assumed to be endemic in 66 districts. From these districts, grouped into strata, 20 districts were randomly selected for M&E in the first sampling stage, with number of districts per province proportional to population: six districts from Western Province, three from the Rift Valley, five from the Coast, and six from Nyanza (Table 1). At the second sampling stage, primary schools were randomly selected from within the chosen 20 districts.

**Table 1:** Number of schools to be sampled in each province for each tier of monitoring

**Province**	**National baseline**	**Pre-post**	**High-frequency**	**Totals**
Western	39	18	3	**60**
Rift Valley	19	9	1	**30**
Coast	33	15	4	**50**
Nyanza	39	18	2	**60**
**Totals**	**130**	**60**	**10**	**200**

### Ethical approval

Ethics approval for this study was granted by the KEMRI Ethics Review Committee in Kenya.

### Data collection

The baseline surveys were conducted between January and April 2012. In each school, 18 children (9 girls and 9 boys) were sampled randomly from each of six classes - one Early Childhood Development (ECD) class and classes 2–6 - using computer generated random number tables, for a total of approximately 108 per school. The sampling within these specified classes aimed to target children aged 5–16 years. Stool samples were obtained for each child and two slides prepared and examined for the presence and intensity of STH species and *S. mansoni* using the Kato Katz method, with the concentration of eggs expressed as eggs per gram (epg) of faeces. Urine samples were obtained only from children in Coast Province (where *Schistosoma haematobium* is widespread) and investigated for presence and intensity of *S. haematobium* using the urine filtration method, with the concentration of *S. haematobium* eggs estimated in eggs per 10 ml urine. Egg counts were performed only up to 24,000 epg and 1,000 eggs/10 ml urine, respectively. Infection intensities above these values were, therefore, not further quantified. In the 10 “high frequency” schools, finger-prick blood samples were obtained and analysed using a HemoCue photometer (HemoCue, Angelhom, Sweden) to estimate haemoglobin concentration. The geographical coordinates of schools were collected using an eTrex global positioning system (Garmin Ltd., Olathe, KS, US).

### Geographic data

Survey data were linked by school location and mapped using ArcGIS 9.3.1 (Environmental Systems Research Institute Inc. Redlands, CA, US). A set of ecological and climatic covariates were assembled from a variety of sources and linked to school locations. Estimates of monthly mean temperature and annual precipitation at 1 km resolution were obtained from WorldClim [11] and elevation data at 100 m resolution were obtained from the Consortium for Spatial Information [12]. Monthly mean, maximum and minimum NDVI estimates at 1 km resolution for 2011 were retrieved from the SPOT Vegetation Programme [13] at a resolution of 1 km. Location of rivers and water bodies were obtained from the Digital Chart of the World [14] and Euclidean distances were calculated at 50 m resolution. Population density estimates at 1 km resolution were obtained from the Afripop project [15] and land cover information from the GlobCover project [16] at a 300 m resolution.

### Statistical analysis

Observed prevalence of each STH species was calculated at school, district and province levels, and 95% confidence intervals (95% CI) were obtained by binomial logistic regression, taking into account clustering by schools. Comparisons of prevalence by location, age group and sex were tested for significance on the basis of the Wald test. For the purposes of analysis, the following age groups were used: 3–5, 6–7, 8–9, 10–11, 12–13, and >14 year-olds. Mean egg counts were expressed as arithmetic mean epg and since egg counts are not normally distributed, 95% CIs were estimated using a negative binomial regression model taking into account clustering. Anaemia was defined using age- and sex-corrected WHO thresholds [17], adjusted by altitude [18]. Infection intensities were further classified into moderate-heavy infections according to WHO guidelines [2] and the prevalence of moderate-heavy infections and 95% CIs were calculated by binomial logistic regression taking into account clustering. Ninety five percent CIs of mean haemoglobin concentrations and prevalence of anaemia were obtained by linear and binomial regression analysis, respectively, both taking into account clustering by schools. The associations of any or moderate-heavy infections with anaemia were investigated by binomial regression analysis adjusting for age and sex of children and taking into account clustering. Comparisons were tested for significance using the Wald-test following regression analysis.

The presence of spatial autocorrelation in prevalence data was investigated using Moran’s I statistic. Moran’s I coefficient of autocorrelation is a measure of similarity of outcome variables among spatially related areas, using a weighted matrix to define spatial relationships. A coefficient of 0 indicates the null hypothesis of no clustering, while a positive coefficient indicates positive spatial autocorrelation [19]. Therefore, large-scale spatial effects were first removed using binary logistic regression models that modelled prevalence as a function of survey location and environmental characteristics. To reduce the number of variables and to prevent multi-collinearity, variables were first subjected to factor analysis. Five factors with Eigenvalues greater than 1 were retained: factor 1 was mainly determined by mean, minimum, maximum temperature and altitude, factor 2 by Euclidean distance to any and permanent water bodies, factor 3 by mean and maximum NDVI, factor 4 by distance to any and permanent rivers, and factor 5 by population density and population density < 5 year-olds. Factor values for each school were predicted and included into the binomial regression models. Land cover, NDVI standard deviation, and annual precipitation did not strongly contribute to these factors and were, therefore, included additionally into the models. The resultant normally distributed Pearson residuals from the regression analysis were used to estimate the Moran’s I statistic. All statistical analyses were carried out using STATA version 12.0 (STATA Corporation, College Station, TX, US).

## Results

Overall, data were collected from 21,528 children, including 4,944 (23.0%) from Coast Province, 3,658 (17.0%) from Rift Valley Province, 6,018 (28.0%) from Western Province, and 6,908 (32.1%) from Nyanza Province (Table 2). Errors in recording information in the field meant that information on age and sex of children were available for 21,312 children (99.0%) and 21,342 children (99.1%), respectively. The mean age of children was 9.8 years (standard deviation, SD 2.8 years) and the age range was 3 to 21 years. The percentage of boys (49.9%) and girls (49.2%) was comparable.

**Table 2:** Prevalence and intensity of infections, by province and range of school-level prevalence

**Province/species**	**N examined**	**N infected**	**% infected (95%CI**^**1**^**)**	**School prevalence range (%)**	**Mean epg (95%CI**^**2**^**)**
**Coast**					
Hookworm	4944	898	18.2 (14.0- 23.5)	0-59.3	64 (34–121)
*A. lumbricoides*	4944	51	1.0 (0.7-1.6)	0-8.3	29 (15–54)
*T .trichiura*	4944	390	7.9 (5.7-10.9)	0-44.4	10 (5–21)
**Rift Valley**					
Hookworm	3658	129	3.5 (2.1 -6.0)	0-21.9	18 (6–57)
*A. lumbricoides*	3658	992	27.1 (21.9-33.5)	0-55.6	3445 (2707–4385)
*T. trichiura*	3658	435	11.9 (7.7-18.3)	0-64.4	31 (17–57)
**Western**					
Hookworm	6018	1,513	25.1 (21.4-29.5)	0-57.9	144 (116–177)
*A. lumbricoides*	6018	1459	24.2 (20.4-28.7)	0.9-63.0	1616 (1293–2020)
*T. trichiura*	6018	351	5.8 (3.8-8.8)	0-40.7	17 (10–28)
**Nyanza**					
Hookworm	6908	812	11.8 (9.8-14.1)	0-35.2	19 (14–26)
*A. lumbricoides*	6908	1373	19.9 (16.0-24.7)	0-71.3	1898 (1356–2655)
*T. trichiura*	6908	250	3.6 (2.6-5.1)	0-26.9	64 (10–399)
**Total**					
Hookworm	21528	3352	15.6 (13.7-17.7)	0-59.3	64 (51–81)
*A. lumbricoides*	21528	3875	18.0 (15.7- 20.6)	0-71.3	1653 (1372–1991)
*T. trichiura*	21528	1426	6.6 (5.4- 8.1)	0-64.4	33 (10–104)

^1^Confidence intervals obtained by binomial regression taking into account school clusters.

^2^Confidence intervals obtained by negative binomial regression taking into account school clusters.

### Soil-transmitted helminths

Overall, 32.4% (95% CI 30.1-34.8%) were infected with at least one STH species. *A. lumbricoides* was the most prevalent STH species (18.0%, 95% CI 15.7- 20.6%), followed by hookworm (15.6%, 95% CI 13.7-17.7%) and then *T. trichiura* (6.6%, 95% CI 5.4- 8.1%). The overall mean intensity of *A. lumbricoides* was 1,653 epg (95% CI 1372–1991), mean hookworm intensity was 64 epg (95% CI 51–81) and mean *T. trichiura* intensity was 33 epg (95% CI 10–104).

**Figure 2:**
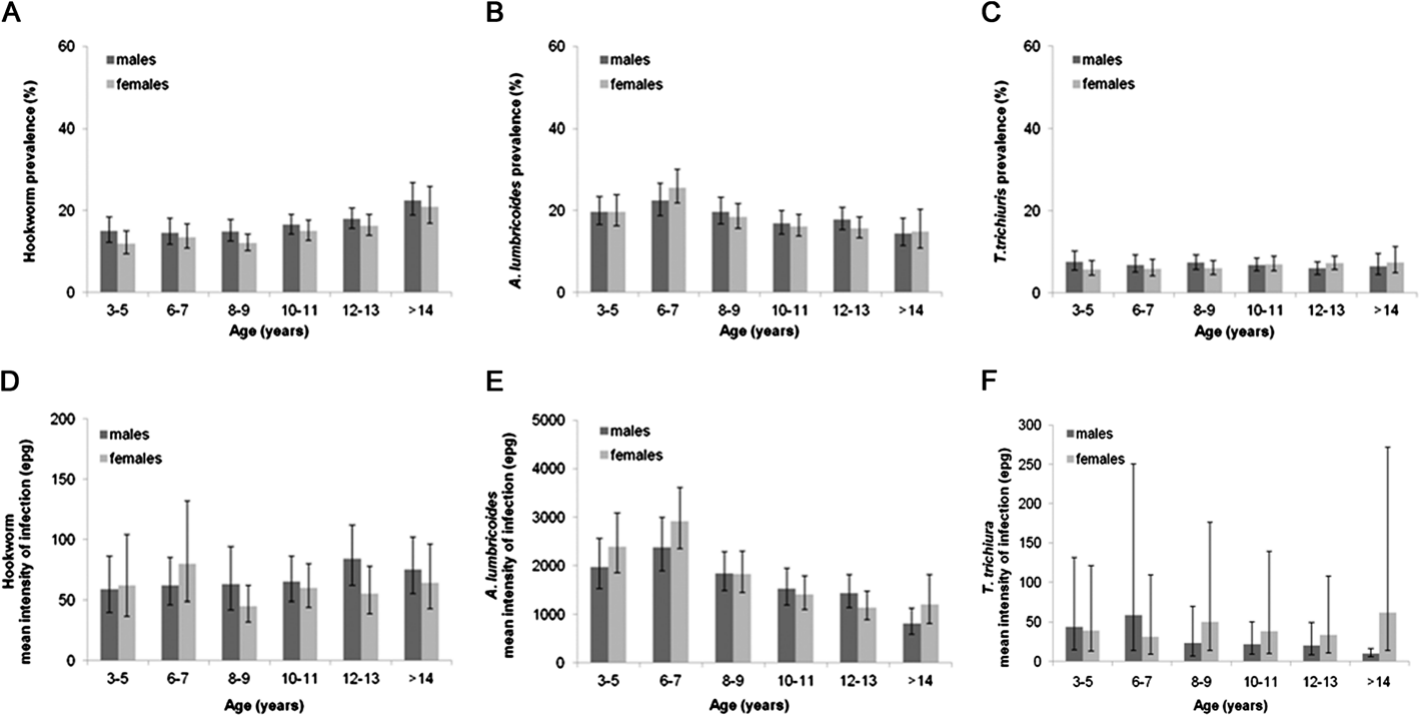
**Prevalence of infection and mean intensity of infection by age and sex for hookworm (A, D), *A. lumbricoides *(B, E), and *T. trichiura *(C, F).** 95% Confidence intervals were obtained by binomial regression and negative binomial regression, respectively taking into account school clusters.

Overall, the prevalence and intensity of hookworm was higher in boys than girls (16.8%, 95% CI 14.8-19.0 vs. 14.5%, 95% CI 12.6-16.7, p < 0.001, 70 epg, 95% CI 55–88 vs. 58 epg, 95% CI 44–76, p = 0.015). Prevalence of *A. lumbricoides* and *T. trichiura* did not differ significantly by sex (p = 0.527 and p = 0.701). The intensity of *T. trichiura* infection was lower among boys than girls (27 epg, 95%CI 10–74 vs 40 epg, 95% CI 11–139, p = 0.002), while the intensity of *A. lumbricoides* did not differ significantly by sex (p = 0.310). Figure 2 presents prevalence and intensity of infection by age and sex. For both boys and girls, the prevalence of hookworm varied significantly by age group (p < 0.001), with prevalence highest among >14 year-olds; however, hookworm intensity did not vary significantly by age group (p = 0.250 and p = 0.051). Both the prevalence and intensity of *A. lumbricoides* varied significantly by age (p < 0.001), among boys and girls, and was highest among 6–7 year-olds. The prevalence of *T. trichiura* did not vary by age group (p = 0.355 for boys and p = 0.284 for girls), whereas the intensity of *T. trichiura* did vary significantly by age group (p < 0.001), with intensity being highest among 3–7 year-old male children and 8–9 and >14 year-old female children.

**Figure 3:**
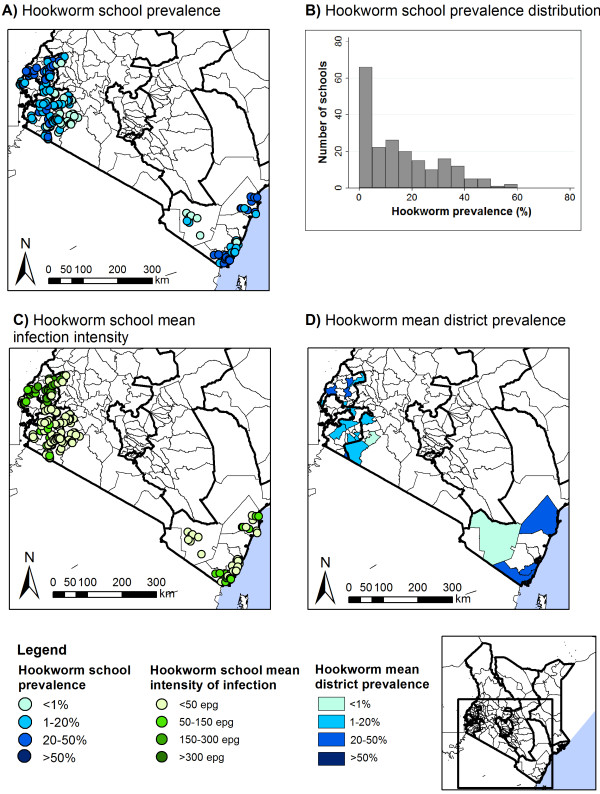
**Spatial distribution of hookworm prevalence and intensity, by school and district.** Spatial distribution of hookworm school prevalence (**A**) and average school infection intensity (**C**), school prevalence distribution (**B**), and average district prevalence (**D**).

**Figure 4:**
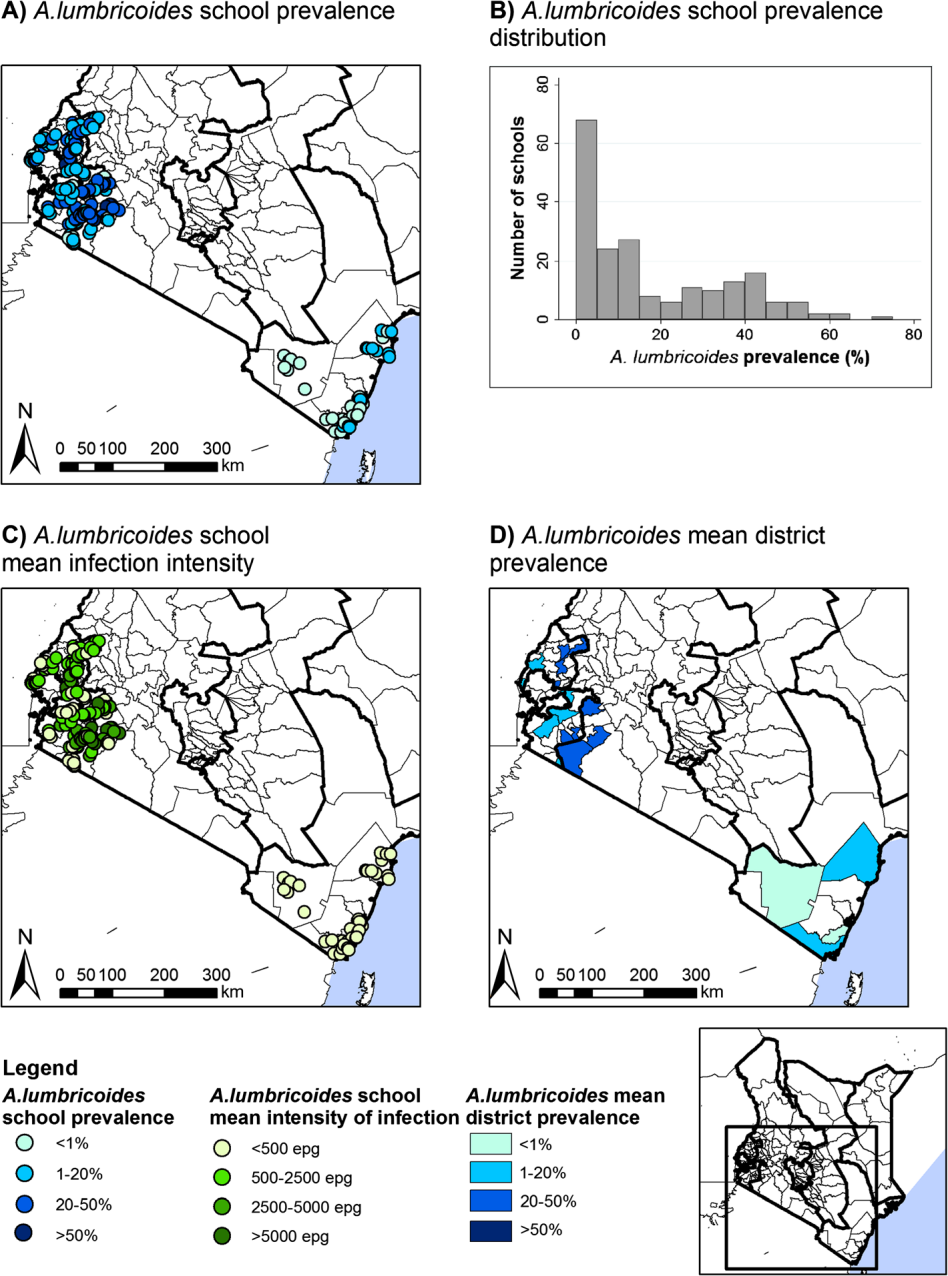
**Spatial distribution of *A. lumbricoides *prevalence and intensity, by school and district.** Spatial distribution of *A. lumbricoides* school prevalence (**A**) and average school infection intensity (**C**), school prevalence distribution (**B**), and average district prevalence (**D**).

**Figure 5:**
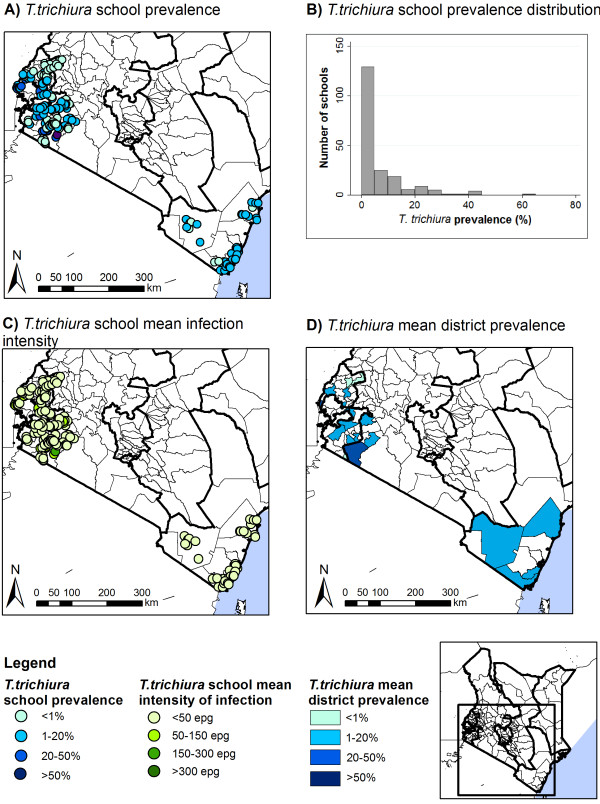
**Spatial distribution of *T. trichiura *prevalence and intensity, by school and district.** Spatial distribution of *T. trichiura* school prevalence (**A**) and average school infection intensity (**C**), school prevalence distribution (**B**), and average district prevalence (**D**).

Table 2 summarizes the prevalence and intensities of STH infection by province. Patterns of STH varied markedly by province (p < 0.001), with hookworm most prevalent in Western Province and *A. lumbricoides* and *T. trichiura* most common in Rift Valley Province. Intensities of hookworm and *A. lumbricoides* infection also varied by province (p < 0.001), but there was only suggestive evidence for a difference in *T. trichiura* intensity by province (p = 0.055). The prevalence of moderate-heavy infection intensities was highest for *A. lumbricoides* in the Rift Valley (15.7%, 95% CI: 12.4-19.9, p < 0.001) and for hookworm in the Coast and Western Proince (0.5%, 95% CI: 0.2-1.1 and 0.5, 95% CI: 0.3-0.9, p < 0.001). The overall prevalence of moderate-heavy *T. trichiura* infections was 0.3% (95% CI: 0.2-0.6, p = 0.133). Figures 3,4,5 present the geographical variation of prevalence and intensity of infection by school and by district. Prevalence of infection varied markedly by school across the country: *A. lumbricoides* ranged from 0–71.3%; hookworm from 0–59.3%, *T. trichiura* from 0–64.4% (Table 2 and Figures 3A-5A). Prevalence by district varied from 0.3-45.5%, 0.2-44.3% and 0.2-30.2%, respectively (Figures 3D-5D). After removal of large-scale environmental effects, small-scale spatial patterns of prevalence, as indicated by Moran’s I statistic of spatial autocorrelation could be observed for hookworm (p = 0.045, p < 0.001) and *A. lumbricoides* (p < 0.001) in all regions, and for *T. trichiura* in Western, Rift Valley and Nyanza Provinces (p = 0.008) (Table 3).

**Table 3:** **Moran’s I statistic (I) of hookworm, *A. lumbricoides*, *T.trichiura*, S. mansoni, and *S. haematobium *prevalence spatial autocorrelation after removal of large-scale environmental effects**

	**Coast**		**Western/Rift Valley/ Nyanza**	
	**I**	**p-value**	**I**	**p-value**
Hookworm	0.118	<0.045	0.168	<0.001
*A. lumbricoides*	0.432	<0.001	0.121	<0.001
*T. trichiura*	0.092	0.077	0.067	0.008
*S. mansoni*	0.221	0.002	0.030	0.113
*S. haematobium*	−0.055	0.414	NA	NA

### Schistosome infection

The overall prevalence of *S. mansoni* was 2.1% (95% CI 1.2- 3.5) and the mean infection intensity was 12 epg (95%CI 4–36). In the Coast Province, where urine samples were collected, the prevalence of *S. haematobium* was 14.8% (95% CI 11.3-19.5) and the mean infection intensity was 16 eggs/10 ml urine (95% CI 10–26).

**Figure 6:**
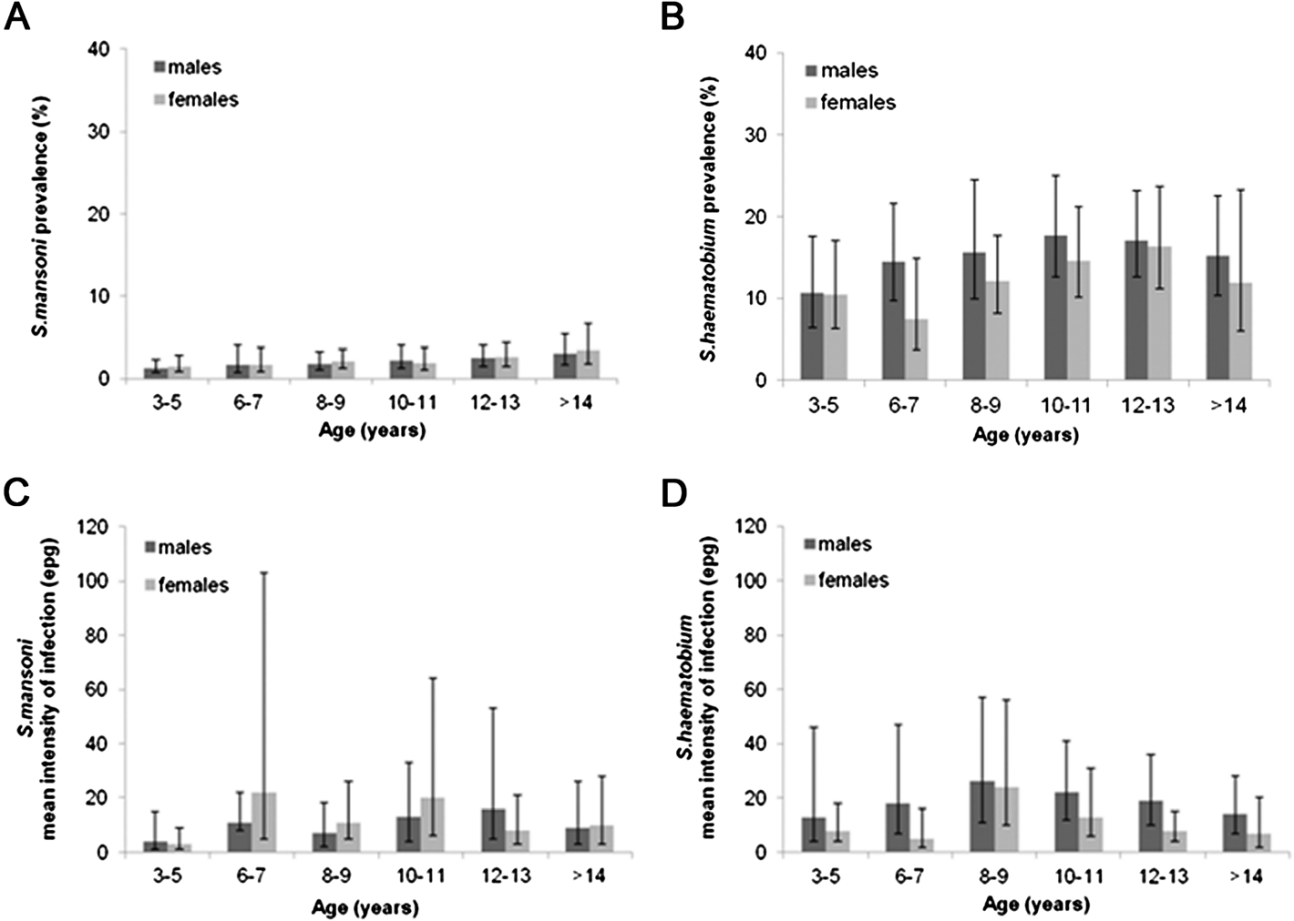
***S. mansoni *and *S. haematobium *prevalence (A-B) and mean infection intensity (C-D) by age and sex.** 95% Confidence intervals of prevalence were obtained by binomial regression and confidence intervals of infection intensity by negative binomial regression, both taking into account school clusters.

There was no significant difference by sex in the prevalence of *S. mansoni* (p = 0.868), prevalence of *S. haematobium* or intensity of *S. mansoni* (p = 0.362), whereas the intensity of *S. haematobium* was higher among boys than girls (32 eggs/10 ml, 95%CI 14–74 vs 12 eggs/10 ml, 95%CI 7–20, p = 0.033). *Schistosoma* infection prevalence and intensity by age and gender is shown in Figure 6. The prevalence (p_linear_ = 0.001 and p_linear_ = 0.018) and intensity (p < 0.001 and p = 0.031) of *S. mansoni* varied significantly by age-group among both sexes. While prevalence increased linearly with age, intensity of infection was lowest among 3–5 year-olds. The evidence for a variation in *S. haematobium* prevalence or intensity by age-group was weak among boys and girls (prevalence p = 0.308, p = 0.066; intensity: p = 0.640, p = 0.265).

**Figure 7:**
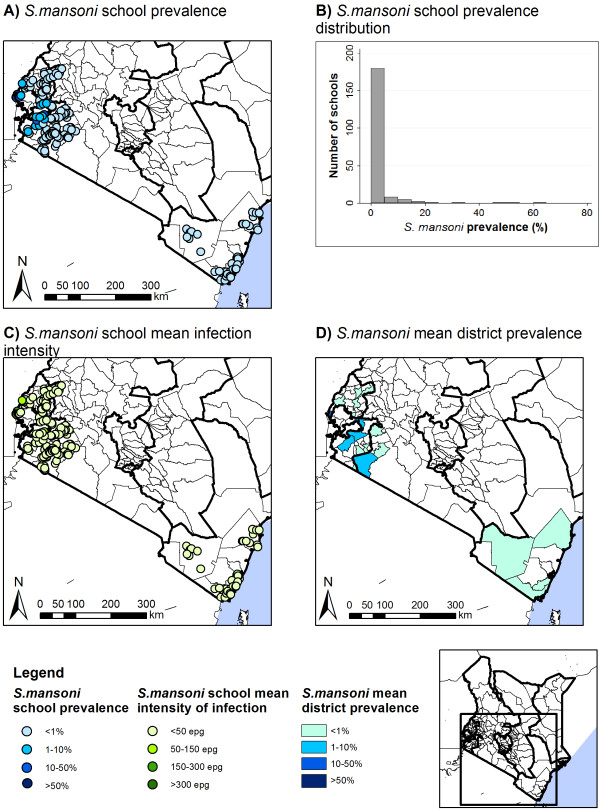
**Spatial distribution of *S. mansoni *prevalence and intensity, by school and district.** Spatial distribution of *S. mansoni* school prevalence (**A**) and average school infection intensity (**C**), school prevalence distribution (**B**), and average district prevalence (**D**).

**Figure 8:**
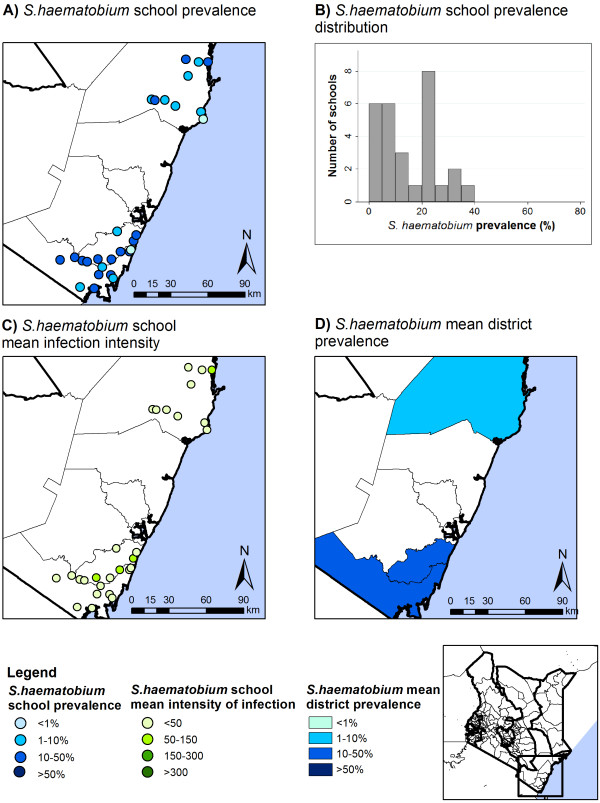
**Spatial distribution of *S. haematobium *prevalence and intensity, by school and district.** Spatial distribution of *S. haematobium* school prevalence (**A**) and average school infection intensity (**C**), school prevalence distribution (**B**), and average district prevalence (**D**).

Prevalences and intensities of *Schistosoma* infections are summarized in Table 4. *S. mansoni* infection prevalence and intensity varied by provinces (p < 0.001) and both were highest in Western Province. The prevalence of moderate-heavy *S. mansoni* infection intensity was also highest in Western Province (3.4%, 95% CI: 1.5-8.1. p < 0.001). There was also marked variability by school and district: school prevalence ranged from 0–64.8% for *S. mansoni* and 0-37% for *S. haematobium*; and mean district prevalences were 0–27.5% and 10-23%, respectively (Figures 7 and 8).

**Table 4:** **Prevalence and intensity of *Schistosoma mansoni *and *S. haematobium, *by province and variation by school**

	**N examined**	**N infected**	**% infected (95%CI**^**1**^**)**	**School prevalence range (%)**	**Mean epg (95% CI**^**2**^**)**
**Coast**					
*S. mansoni*	4944	1	0.0 (0.0-0.1)	0-0.9	0 (0–0)
*S. haematobium*	3019	448	14.8 (11.3-19.5)	0-37.0	16 (10–26)
**Rift Valley**					
*S. mansoni*	3658	13	0.4 (0.1-2.5)	0-12.0	1 (0–9)
**Western**					
*S. mansoni*	6018	246	4.1 (1.9-9.0)	0-64.8	40 (12–126)
**Nyanza**					
*S. mansoni*	6908	190	2.8 (1.5-5.0)	0-47.2	3 (1–7)
**Total**					
*S. mansoni*	21528	450	2.1 (1.2-3.5)	0-64.8	12 (4–36)

^1^95%CIs obtained by binomial regression taking into account school clusters.

^2^95%CIs obtained by binomial regression taking into account school.

After removing environmental large-scale effects, positive spatial autocorrelation was observed only for *S. mansoni* prevalence in the Coast Province (p = 0.002), while the evidence for small-scale spatial structures was weak for *S. mansoni* in the other areas (p = 0.113) or for *S. haematobium* (p = 0.414). Moran’s I statistics of spatial autocorrelation are summarised in Table 3.

### Anaemia

Finger-prick blood samples were collected from 492 children from 10 schools. Although too small to be meaningfully representative of the country, these ten schools were chosen to include schools in each of the four provinces where STH endemicity had been predicted. In these ten schools, the overall prevalence of anaemia was 31.3% (95% CI 21.7-45.1%) and the overall mean haemoglobin concentration was 12.6 g/dL (95% CI 12.0-13.2 g/dL). No significant differences were found in anaemia and haemoglobin concentration by sex (anaemia: p = 0.264; haemoglobin: p = 0.947), but there was significant variation by age (anaemia: p = 0.046; haemoglobin: p = 0.048), with anaemia decreasing and haemoglobin increasing with increasing age.

The overall prevalence of STH infection in these 10 schools was 33.3% (95% CI 25.7-43.1%, range of school prevalence: 18.5-65.7%) and the prevalence of schistosome infection was 14.3% (95% CI 7.1-28.7%, range of school prevalence: 0–34.3%). There was suggestive evidence for an association of anaemia with *S. mansoni* infection (p = 0.052) after adjusting for age and sex of children. However, there was no strong evidence for a positive association of anaemia with any or moderate-heavy hookworm, *A. lumbricoides*, *T. trichiura*, or *S. haematobium* infections and moderate-heavy *S. mansoni* infections.

## Discussion

This survey provides an up-to-date assessment of STH infections in the regions of Kenya targeted for school-based deworming, and provides a rigorous basis for evaluating programme impact. The most common STH species detected among children was *A. lumbricoides,* followed by hookworm and *T. trichiura*. The prevalence and intensity of species infection varied markedly at province, district and schools levels. At an individual level, patterns of infection and intensity varied by age-group and sex of the children.

In Kenya, STHs are assumed to be endemic in 66 districts that were identified based on historical data and predictive maps created using a Bayesian space-time geostatistical modelling approach [9,10]. When comparing the predictions with the survey data, two districts that were suggested to require MDA have an actual mean STH prevalence < 20%. These are Kisumu East District in Nyanza Province (17.4%) and Kilindini in Coast Province (18.0%). However, the ranges of school prevalence in these districts show spatial heterogeneity and schools in western parts of Kisumu and in the North of Kilindini exceed indeed the threshold prevalence. A deviation from the prediction can be observed for Transmara District in Rift Valley Province. While the predictive map suggested prevalence between 10 and 20% and no intervention was recommended, the district has the highest mean district STH prevalence in this study (53.1%). It is noteworthy that no historical survey data were previously available for this district. Furthermore, the newly obtained survey data should be used to revise the predictions, especially for areas with previous high uncertainty.

Interestingly, when comparing the baseline survey results to historical data, STH infection prevalence has strongly decreased over the last decade in certain regions of Kenya. These are specifically Bunyala District, Western Province, where a deworming programme has been implemented since 1998 [20,21] and Kwale District, Coast Province, where the National Programme for Elimination of Lymphatic Filariasis led to four rounds of Diethylcarbamazine citrate (DEC) and albendazole distribution [22,23]. In other districts where chemotherapy programmes have not been implemented previously, such as Homa Bay in Nyanza Province, the prevalence estimates did not change over time [24-32].

After removal of large scale environmental effects, small-scale spatial patterns could be observed for hookworm and *A. lumbricoides* in all regions and *T. trichiura* in Western, Rift Valley and Nyanza Provinces. Previous studies in Kenya, Uganda, Tanzania, and Zambia showed spatial correlation up to 95–166 km for hookworm and 36–92 km for *A. lumbricoides*[33,34]. The lack of spatial correlation of *T. trichiura* was reported in several other studies, although Sturrock *et al*. were able to show spatial dependency up to a distance of 46 km in Kenya’s Coast Province [33,34]. Nevertheless, the spatial autocorrelation p-value for Coast Province in this study is quite small (p = 0.077), and might still be indicative of a certain spatial structure of *T. trichiura* infection. The observed small-scale patterns of infections might originate from specific characteristics of the locations that influence the risk of infection, such as access to water, sanitation and hygiene (WASH) [35,36].

Even though the overall prevalence of *S. mansoni* was low, prevalence reached up to 64% in some schools in Western Province and up to 47.2% in schools in Nyanza Province. This is consistent with data collected between 1980 and 2009, showing small levels of infection in coastal regions and higher prevalence near the shores of Lake Victoria [10,37]. Previously collected data for *S. haematobium* infections, however, show higher prevalence than actually observed in this survey [10]. This highlights that historical data can serve as an indicator for endemic regions, however, programme implementers need to be aware of a certain degree of uncertainty. As for STH infections, *S. mansoni* infection varied significantly between and within provinces. Additionally, small-scale spatial patterns of *S. mansoni* were observed within Coast Province, which is consistent with studies in Cameroon, Mali, and Uganda, where spatial correlation ranged up to 70 km [38]. For Western, Nyanza, and Rift Valley Provinces, significant evidence for spatial autocorrelation was observed only before removal of environmental effects. Interestingly, there was no spatial pattern for *S. haematobium* prevalence before or after de-trending the data. This is surprising, as studies from Tanzania and Sierra Leone demonstrated autocorrelation of *S. haematobium* infections [39,40]. Nevertheless, infection prevalence was associated with proximity to water bodies and annual rainfall.

The main limitation of this study is the lack of a control group to evaluate the effect of the deworming programme. As the deworming programme is implemented nationally and the ministries of health and education plans to deworm all children in the 66 endemic districts, an exclusion of groups of children from this effort would be unethical. As compensation for this constraint, series of pre-post and high-frequency samplings are performed, which allow a more direct attribution of reductions in infection to programme implementation and help to distinguish treatment effects from any other time-varying fluctuations in infection rates. A challenge experienced in the field was the use of paper questionnaires to collect data and in rare instances there was inaccurate recording of age and sex. To overcome this issue, future data collection intends to use electronic data collection. A further study limitation is the use of a single stool sample for Kato-Katz analysis and the truncation of egg counts at a certain maximum level for the measurement of infection intensities. The sensitivity of Kato-Katz analysis might be lower for the detection of light infections than for example formol-ether concentration techniques and could have been improved by processing more than one sample [41]. This potentially leads to lower than actually detected infection prevalence and mean infection intensities. Finally, because anaemia data were only collected from ten schools, we cannot make generalizations across the entire country, and the small sample makes associations with helminth infection difficult to assess. Nonetheless, there was evidence of an association between *S. mansoni* infection and the risk of anaemia.

## Conclusions

The analysis of collected data provided insight into the current prevalence and distribution of spatial patterns of STH and schistosome infections in Kenya and will allow monitoring of the impact of the national deworming programme on the prevalence and intensity of infection and on child health outcomes. Furthermore, areas of high prevalence of schistosome infection that merit mass drug administration were identified, while other areas should be further assessed for *S. haematobium* infections. Additional studies using individual data on potential risk factors of infections might enable us to explain the observed small-scale spatial patterns of infection and provide further insights into determinants of STH and schistosome infections in Kenya. Monitoring the impact of school-based deworming using these baseline findings will allow the Government of Kenya to make informed decisions on the allocation of resources and on what strategies to adopt when infection-level has significantly dropped.

## Competing interests

The authors declare that they all have no competing interests.

## Authors’ contributions

CSM, OO and SMN designed the study, with support from AH, RLP and SJB. JHK coordinated the data collection and DAM and MTM provided parasitological expertise. BN conducted the data analysis. CSM, BN and SJB wrote the first draft and all authors read and approved the final version of the manuscript. All authors read and approved the final manuscript.
